# Using data from cue presentations results in grossly overestimating semantic BCI performance

**DOI:** 10.1038/s41598-024-79309-y

**Published:** 2024-11-14

**Authors:** Milan Rybář, Riccardo Poli, Ian Daly

**Affiliations:** Brain-Computer Interfaces and Neural Engineering Laboratory, School of Computer Science and Electronic Engineering, University of Essex, Colchester, CO4 3SQ UK

**Keywords:** Semantic decoding, Mental imagery, Cue presentation, Electroencephalography (EEG), Machine learning, Brain-computer interface (BCI), Functional magnetic resonance imaging (fMRI), Biomedical engineering, Neural decoding, Translational research, Machine learning

## Abstract

Neuroimaging studies have reported the possibility of semantic neural decoding to identify specific semantic concepts from neural activity. This offers promise for brain-computer interfaces (BCIs) for communication. However, translating these findings into a BCI paradigm has proven challenging. Existing EEG-based semantic decoding studies often rely on neural activity recorded when a cue is present, raising concerns about decoding reliability. To address this, we investigate the effects of cue presentation on EEG-based semantic decoding. In an experiment with a clear separation between cue presentation and mental task periods, we attempt to differentiate between semantic categories of animals and tools in four mental tasks. By using state-of-the-art decoding analyses, we demonstrate significant mean classification accuracies up to 71.3% during cue presentation but not during mental tasks, even with adapted analyses from previous studies. These findings highlight a potential issue when using neural activity recorded during cue presentation periods for semantic decoding. Additionally, our results show that semantic decoding without external cues may be more challenging than current state-of-the-art research suggests. By bringing attention to these issues, we aim to stimulate discussion and drive advancements in the field toward more effective semantic BCI applications.

## Introduction

Neuroimaging research has shown the potential for semantic neural decoding, which aims to identify specific semantic concepts an individual is focused on, or thinking of, at a given moment in time from their neural activity^1,2^. Most of these neuroimaging studies use experiments that are designed to cue participants to focus on instances of specific semantic concepts for short periods of time, for example by presenting visual or auditory stimuli. Neural activity is recorded while participants perform mental tasks involving the cued semantic concepts, for example, by silently naming the instance of the concept. This recorded activity is then processed to attempt to differentiate between distinct semantic concepts or categories.

This semantic neural decoding could be used by brain-computer interfaces (BCIs) for communication^3,4^. BCIs enable a degree of direct control over external devices through signals originating from the human brain. There is a need to explore alternative control schemes for BCI-based communicators. Some options that have been explored are based on decoding language-based signals. For example, recent advances in speech decoding BCIs have shown exciting promise for restoring rapid communication^5–10^. The most remarkable results have been achieved using invasive approaches, such as electrocorticography (ECoG). However, it is unclear whether it is possible to translate these results with a similar level of performance to electroencephalography (EEG) (or other non-invasive practical modalities)^11–15^. Additionally, it has been shown that handwriting imagery can be decoded and used for typing purposes^16,17^.

Semantic BCIs could aid communication by allowing direct communication of semantic concepts for people who experience difficulties communicating via other means^18–20^. The majority of semantic decoding studies have used functional magnetic resonance imaging (fMRI) and only a few studies used EEG^1^. Here, we focus on EEG which provides a better ecological validity in comparison with fMRI and is more suitable for non-invasive BCIs.

An important distinction between semantic BCIs and the majority of semantic decoding studies is that in semantic BCIs, users would freely choose and focus on a semantic concept of their choice (from a supported set of recognizable concepts). In other words, in a semantic BCI, there would not be an external cue to guide the participant’s specific choice. Here, we begin with a preliminary step by using external cues but ensuring they are sufficiently separated from the mental tasks.

However, our efforts to translate these findings into a BCI paradigm have been met with significant challenges, even despite leveraging the same mental task as in the literature. Upon closer examination, there was a major difference between our approach and those approaches used in semantic decoding studies.

Because our objective revolves around advancing toward BCIs, we purposely avoid utilizing neural activity recorded during the cue presentation period for decoding purposes. This is because a BCI application allows users to freely communicate information from a choice of two or more options via decoding their brain activity. Consequently, it is not possible, within a BCI context, to know a priori which choice the user wishes to make and, therefore, it is not possible to rely on neural activity related to presentation of a specific cue. In contrast, the majority of semantic decoding studies in EEG have utilized such neural activity to differentiate between distinct semantic categories or concepts (see Table 1). This was typically done in one of two ways: (1) either the cue was present, for example as an image on the screen, while participants perform mental tasks, or (2) the cue presentation and mental task periods were explicitly separated but neural activity recorded during the cue presentation period together with the neural activity recorded during the mental task was used for decoding purposes. Additionally, this issue is mostly likely to be present in fMRI studies due to slow hemodynamic response unless cue presentation and mental task periods were sufficiently separated.

It is imperative to clarify that our intent is not to criticize the methodologies or analyses of prior studies. Rather, our objective is to push the boundaries further and endeavor to bridge the gap between neuroimaging research and practical BCI applications.

In this paper, we demonstrate radically different decoding performance during the cue presentation and mental task periods in two experiments in which we attempt to differentiate between the semantic categories of animals and tools. We use state-of-the-art decoding analyses and further adapt analyses from two EEG-based semantic decoding studies, one of which used the same mental task. While it is possible to decode the semantic category during the cue presentation period, decoders struggled to differentiate between the semantic category when using data recorded only during any of the tested mental tasks. We discuss potential reasons for these difficulties and their implications. By emphasizing this potential disparity between perceived and actual capabilities of semantic BCIs based on purely imaginary mental tasks without any external cue, we aim to stimulate discussion and drive advancements in the field toward more effective semantic BCI applications.

**Table 1:** Semantic decoding studies using EEG from^1^.

Temporal features	Spectral features	Task	Pres. Mod.	Cue present	Ref.
0-700ms	1-30Hz	out-of-category recognition	VAO	Yes	^21^
95-360ms	4.1-18.3Hz	silent naming task	V	Yes	^22,23^
200-700ms (category), 250-500ms (individual words)	1-30Hz	size judgment	AO	Yes	^24^
0-1.2s	optimized for each participant (0.5-55Hz)	passive listening, silent naming task	AO	Yes	^25^
0-1s	0.1-40Hz	remember all six elements presented in a sequence	O	Yes	^26^
0-1s	1-12Hz	out-of-category recognition	A	Yes	^27^
0-700ms	1-30Hz	in-category recognition	V	Yes	^28^
0-3500ms	2-100Hz	semantic judgment	V+O	No	^29^

The column ‘Pres. Mod.’ represents presentation modalities used: visual (V) (image), auditory (A) (spoken word), or orthographical (O) (written word). Multiple letters indicate that multiple modalities were tested separately, while V+O is an image with a corresponding written name. The column ‘Cue present’ refers to whether or not the cue presentation is present in the temporal window used by the semantic decoding model.

## Background

Semantic neural decoding has been explored using a wide range of different neuroimaging methods, experimental designs, and decoding approaches (see a recent systematic literature review^1^).

The majority of semantic decoding studies used fMRI, while a small number of studies used other methods such as EEG, magnetoencephalography (MEG), or invasive approaches. Due to its high spatial resolution, fMRI-based studies typically achieved the highest decoding performance when measured by standard machine learning metrics such as classification accuracy, rank accuracy, or leave-two-out pairwise comparison. However, these metrics do not take into account the time needed to make a decision. When the decoding performance is evaluated in terms of information transfer rate (ITR)^30–32^, fMRI which is affected by a slow hemodynamic delay requires longer times to make a decision (with the lowest times around two seconds for fMRI versus less than half a second for EEG) and thus it achieves lower ITRs in comparison with electrophysiological neuroimaging methods (in a range of 0.02–9.08 for fMRI, 0.21-24.09 for EEG, and 0.09–149.83 for electrophysiological neuroimaging methods including invasive approaches)^1^.

We identified EEG-based semantic decoding studies based on the recent systematic literature review of semantic neural decoding^1^. Table 1 shows the studies that used either EEG alone or in combination with other neuroimaging modalities (primarily MEG). The majority of these EEG-based semantic decoding studies *used neural activity recorded when the cue was present for decoding purposes* (see the “Cue present” column in Table 1).

Even though it has been shown that semantic processing is independent of the stimulus presentation modality^33,34^, several studies reported significantly different decoding accuracies for different stimulus modalities. For instance, an EEG study by Simanova et al.^21^ reported a higher mean classification accuracy when participants were cued by images in comparison with when they were cued by spoken or written words. A MEG-based study by Ghazaryan et al.^35^ reported challenges of semantic decoding from orthographic stimuli in comparison with prior research based on visual stimuli.

## Results

To investigate decoding performance during the cue presentation and mental task periods, we analyzed EEG data from our two experiments in which we attempted to differentiate between the semantic categories of animals and tools.

### Experiments

**Fig. 1:**
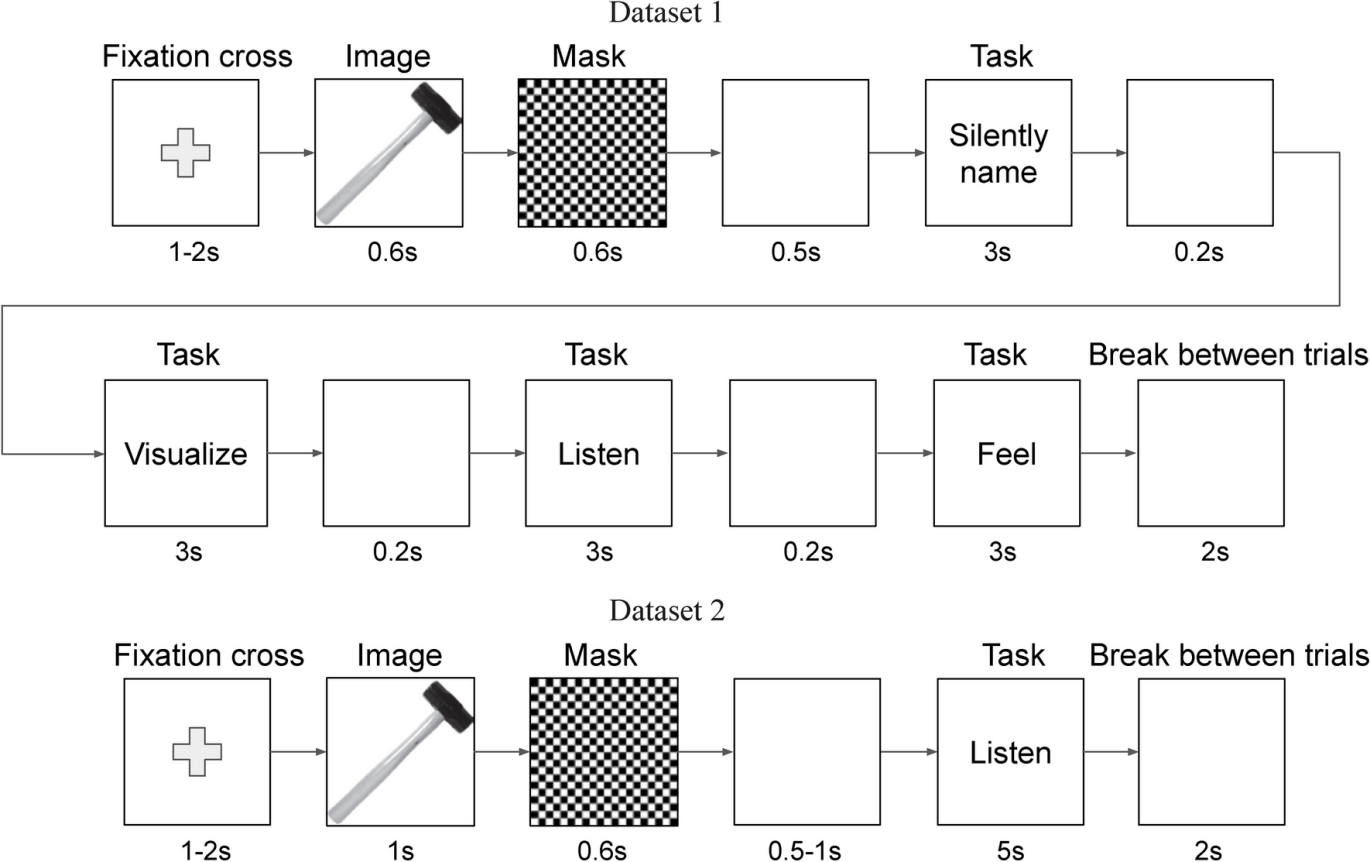
Illustration of one concept trial in Datasets 1 and 2. The order of mental tasks is randomized across blocks in Dataset 1.

In Dataset 1, twelve participants performed four mental tasks: a silent naming task and three imagery sensory-based tasks using visual, auditory, and tactile perception. Participants were asked to visualize an object in their minds, imagine the sounds made by the object, and imagine the feeling of touching the object. Figure 1 shows an illustration of a concept trial. The concept was indicated by an image shown on the screen for 0.6 seconds. A sequence of mental tasks followed after a mask (an image of a checkerboard) which was shown for 0.6 seconds and a black screen which was shown for 0.5 seconds. Each mental task lasted for 3 seconds and was separated from the following one by a blank screen for 0.2 seconds. Each concept was presented five times, that is for a total of 90 trials per category (18 concepts, 5 repetitions each).

In Dataset 2, seven participants performed only the auditory imagery task (see Fig. 1). The cue presentation period of the concept image was extended to 1 second, the duration of the auditory imagery task was extended to 5 seconds, and the blank screen period before the mental task was changed to 0.5–1 second (uniformly distributed). Each concept was presented seven times, that is for a total of 126 trials per category (18 concepts, 7 repetitions each).

### Analysis in the temporal domain

To explore the possibility of differentiating between the semantic categories of animals and tools, we used the most widely used analysis approach from the prior EEG-based semantic decoding studies shown in Table 1 which is an analysis in the temporal domain. This was conducted in the two most widely used frequency bands (see Table 1): 1–30 and 4–30 Hz. Preprocessed EEG data were filtered, downsampled to 128 Hz, and normalized. They were then classified by different classifiers in a stratified cross-validation scheme. We used a support vector machine (SVM) with the radial basis function kernel and $C=1$, logistic regression (LR) with L1 or L2 norm, linear discriminant analysis (LDA), and a random forest (RF) classifier. Additionally, we used the SVM with a value for the *C* parameter chosen by a nested cross-validation, the LR with L1 norm with a regularization strength parameter value selected by a nested cross-validation, and the RF with a nested cross-validation search for a number of trees in the forest and a maximum depth of the tree.

Although, there were differences in decoding performance between the frequency bands of 1–30 and 4–30 Hz, the overall message was the same for both of them. We thus report here only the results using the frequency band of 1–30 Hz to simplify the presentation.

**Fig. 2:**
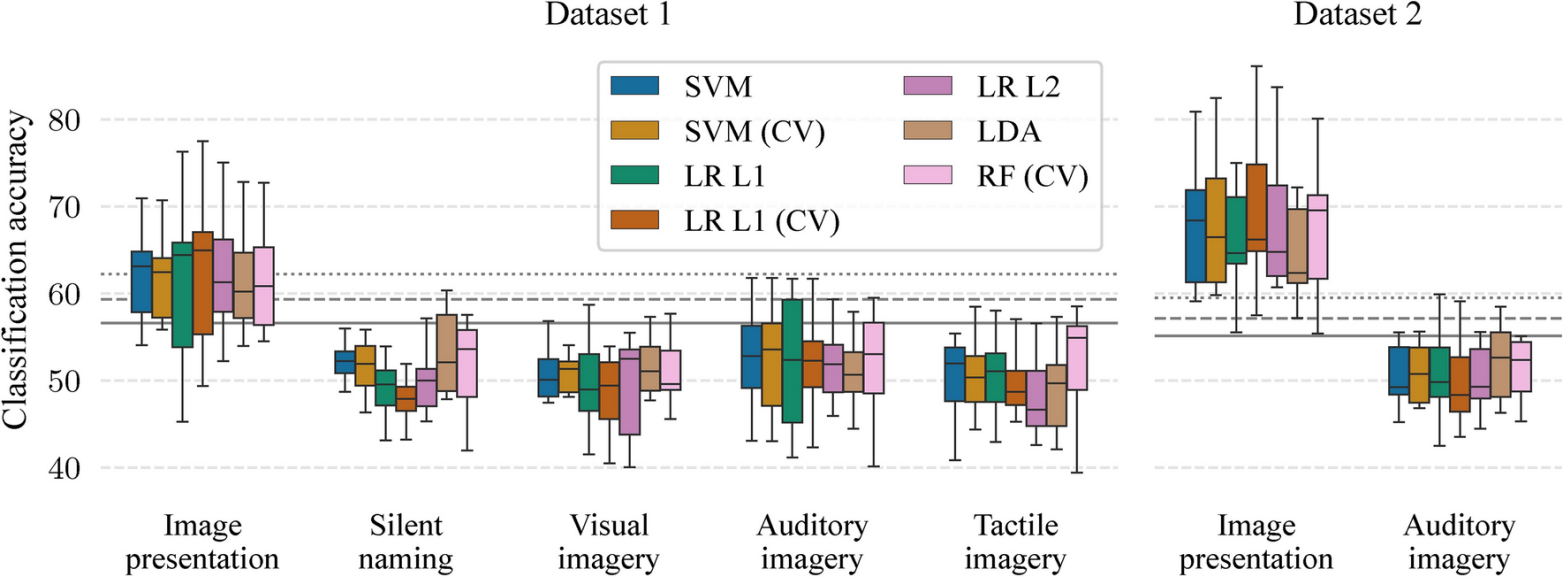
Mean classification accuracies when classifiers can utilize information from all channels and all time points of corresponding periods. Horizontal lines represent significant borderlines for $p=0.05$ (solid), $p=0.01$ (dashed), and $p=0.001$ (dotted).

Figure 2 shows classification accuracies when the classifier can use information from all channels and all time points from the period of interest. It was possible to significantly differentiate between the semantic categories in the image presentation period with mean classification accuracies between 60.4% ($p < 0.01$, a bootstrapping simulation, see Methods) and 62.3% ($p < 0.001$) in Dataset 1 and between 66.9–69.9% ($p < 10^{-6}$) in Dataset 2 across all classifiers. On the other hand, *mean classification accuracies were not significant in any mental task in both datasets*.

A number of participants with individual significant classification accuracies ($p< 0.05$, a bootstrapping simulation) in the image presentation was 8.1 (out of ten participants) on average across all seven classifiers in Dataset 1 while all seven participants had significant accuracies in all classifiers in Dataset 2. These average numbers of participants for the mental tasks were 1.1, 1.0, 2.7, and 1.3 for the silent naming, visual imagery, auditory imagery, and tactile imagery task in Dataset 1, respectively, and 1.0 for the auditory imagery task in Dataset 2.

**Fig. 3:**
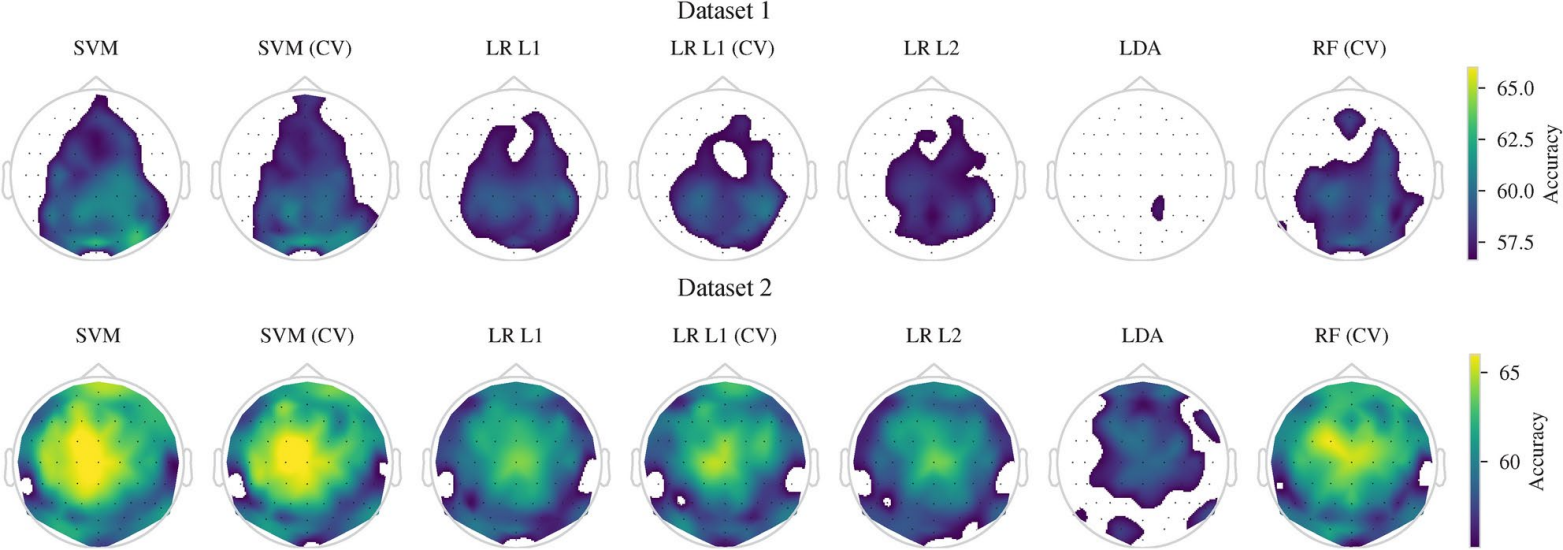
Single-channel mean classification accuracies above the significance borderline ($p = 0.05$) for the image presentation when classifiers can utilize information from all time points of the image presentation period. White represents non-significant classification accuracies.

The percentages of channels with significant single-channel mean classification accuracies ($p < 0.05$) in the image presentation period were between 30.4–58.5% in Dataset 1 and between 51.8–78.9% in Dataset 2. In both datasets, the LDA had the lowest number of significant channels. On the other hand, these percentages for the mental tasks were below 21% in Dataset 1 and below 17.5% in Dataset 2. Figure 3 shows scalp maps with mean classification accuracies for each classifier.

**Fig. 4:**
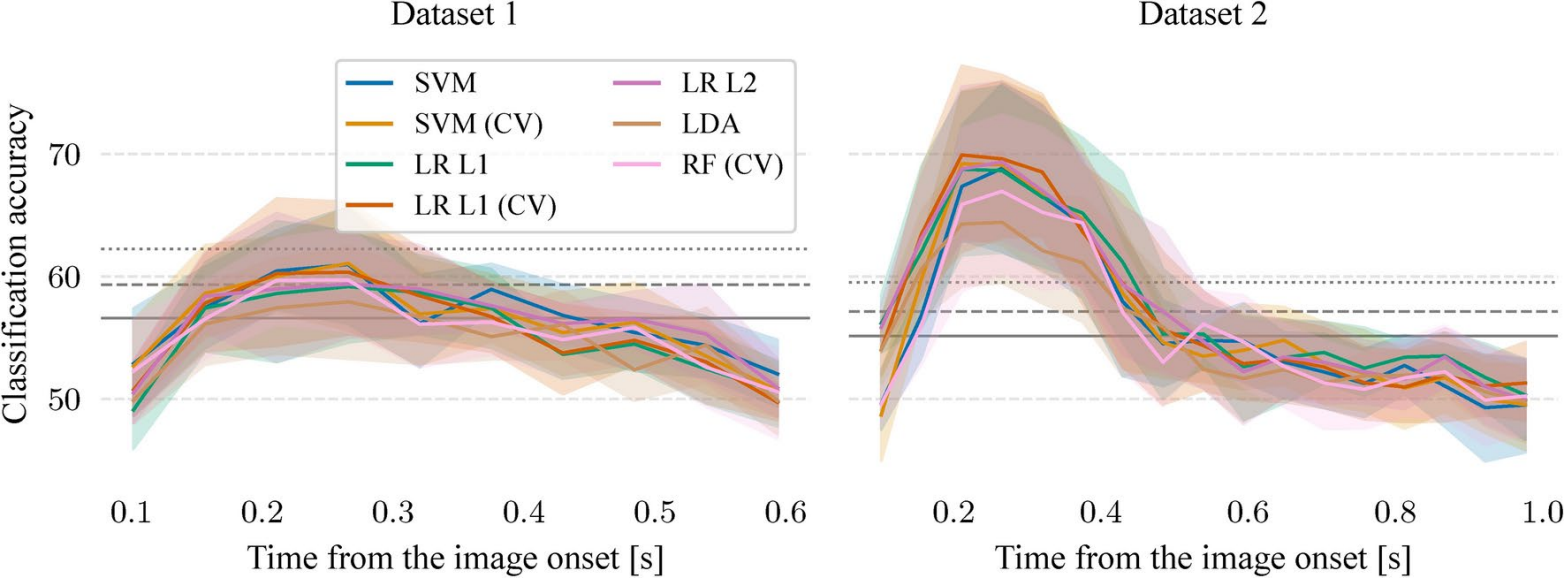
Mean classification accuracies during the image presentation period when the classifier can use information from all channels. Classification accuracies are shown with mean and 95% confidence interval. Horizontal lines represent significant borderlines for $p=0.05$ (solid), $p=0.01$ (dashed), and $p=0.001$ (dotted).

To investigate the temporal evolution of semantic decoding, a sliding window approach was used. A temporal window of 109.375 ms was used (14 time points at a sampling frequency of 128 Hz) with a step of half the window size. A classifier was separately trained in each position of the temporal window by using only time points from that window. Figure 4 shows mean classification accuracies when the classifier can use information from all channels. It was possible to differentiate between the semantic categories during the image presentation period but not during any of the mental tasks in both datasets. The majority of classifiers had significant mean classification accuracies ($p < 0.05$) during the 54.7–429.7 ms period after the image onset (corresponding to the start of the first and the end of the last temporal window) in both datasets. The peak of the mean classification accuracies was in the temporal window of 164.1–265.6 ms after the image onset in both datasets with mean classification accuracies between 57.9% ($p < 0.05$) and 61.1% ($p < 0.01$) in Dataset 1 and between 64.4% ($p < 10^{-5}$) and 69.6% ($p < 10^{-6}$) in Dataset 2.

**Fig. 5:**
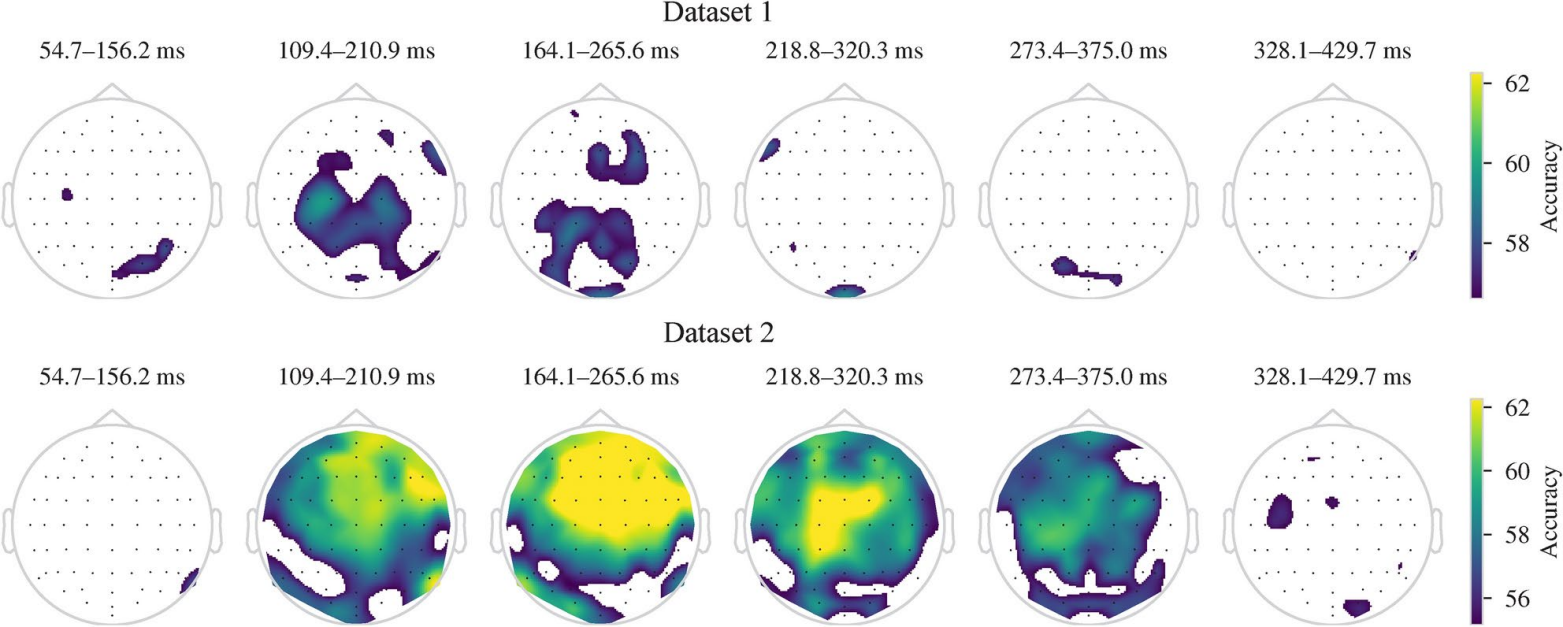
Mean classification accuracies during the image presentation period for each channel using the SVM (CV). Times represent the start and end of each temporal window after the image onset. Scalp maps indicate performance above the significance borderline ($p = 0.05$). White represents non-significant classification accuracies.

Figure 5 shows scalp maps indicating channels for which mean classification accuracies were statistically significant during the image presentation period using the SVM with C parameter selected by nested-cross-validation. The peak of the mean classification accuracies across all participants was again in the temporal window of 164.1–265.6 ms after the image onset in both datasets. Similarly, mean single-channel classification accuracies were not significant during any of the mental tasks in both datasets.

### A comparison to state-of-the-art

To further highlight the need for separating the cue presentation period and the mental task period, we adapted analyses from two studies from Table 1 that used an image stimulus as a cue during the mental task period. It is imperative to clarify again that our intent is not to criticize these studies but to show the potential issue with attempting semantic category decoding using the neural data recorded during the cue presentation period.

In a study by Simanova et al.^21^, participants were presented with either images (line drawings), written words, or spoken words of animals and tools. They were asked to respond, by pressing a mouse button, upon the appearance of items from the non-target task categories (clothing or vegetables). In the case of image stimulus, the image was shown for 300 ms followed by a blank screen for 1000–1200 ms. However, their analysis used a temporal window of 0–700 ms after the stimulus onset which includes the cue presentation period. Filtered signals, in the frequency band 1–30 Hz, from all channels were classified by an LR (Bayesian logistic regression with a multivariate Laplace prior). They achieved a mean classification accuracy of 79% when participants were cued by images. We adapted their analysis by classifying the first 600 ms of the cue presentation period and the mental task periods in the same frequency band. Because our image presentation in Dataset 1 was only 600 ms, in comparison with the 700 ms used by Simanova et al., we used this temporal window of 600 ms for all classified periods.

**Fig. 6:**
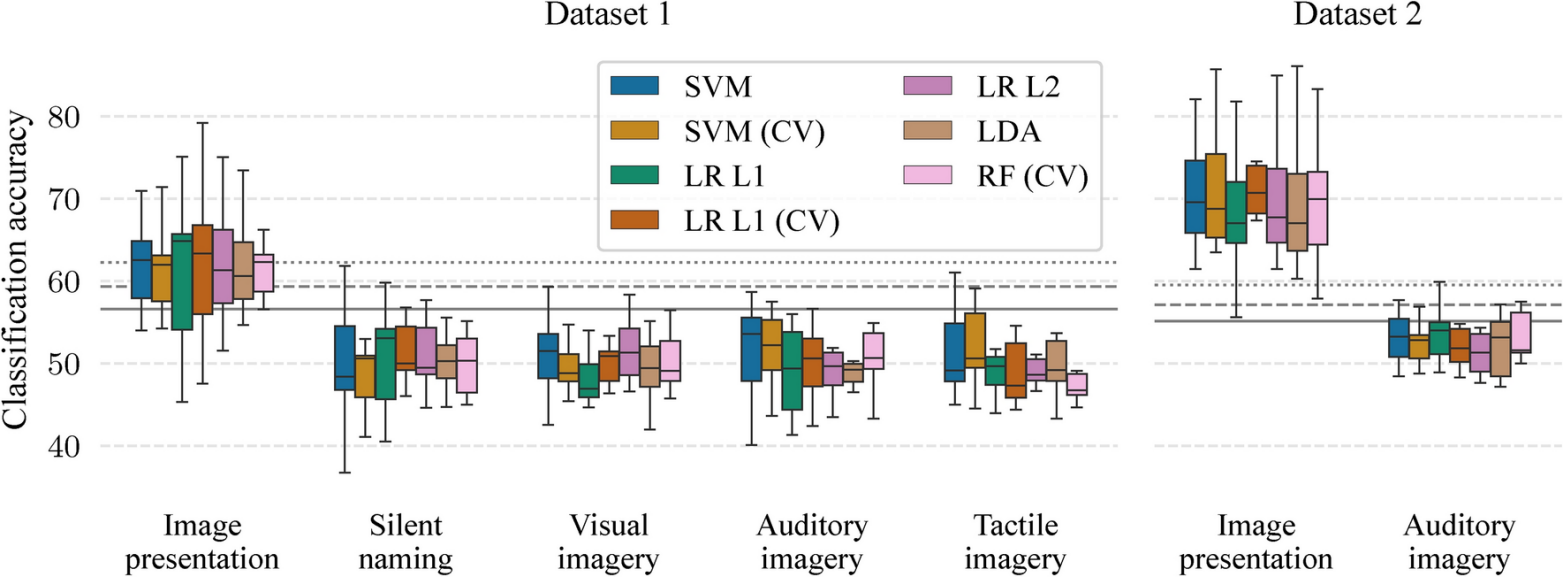
Mean classification accuracies from adapted analysis^21^ in which classifiers can utilize information from all channels and the first 600 ms after the period onset. Horizontal lines represent significant borderlines for $p=0.05$ (solid), $p=0.01$ (dashed), and $p=0.001$ (dotted).

Figure 6 shows the results of this adapted analysis, which used all channels and 600 ms periods. Mean classification accuracies for the image presentation were between 60.2–62.2% ($p < 0.01$) for tested classifiers in Dataset 1 and between 68.2–71.3% ($p < 10^{-6}$) in Dataset 2. Moreover, individual classification accuracies were significant for all participants in Dataset 2 ($p < 0.05$ for the LR with L1 norm, $p < 0.01$ for the LR with L1 norm and nested-cross-validation, and $p < 0.001$ for the rest). For any of the mental task periods, mean classification accuracies were not statistically significant in both datasets. The average number of participants with significant accuracies in the image presentation was 8.4 in Dataset 1 while all participants had significant accuracies in all classifiers in Dataset 2. These average numbers of participants for the mental tasks were 1.0, 1.1, 0.6, and 1.3 for the silent naming, visual imagery, auditory imagery, and tactile imagery task in Dataset 1, respectively, and 1.6 for the auditory imagery task in Dataset 2.

In a study by Murphy et al.^22^, participants were presented with images of mammals and tools. They were asked to silently name the shown object and to press a mouse button when finished (with a timeout of 3 seconds). The image was shown for the whole task duration. Their analysis used a temporal window of 95–360 ms (after the task (or image) onset) and a frequency window of 4.1–18.3 Hz to extract epochs that were then spatially filtered by common spatial patterns (CSP)^36,37^. A subset of *N* CSP components (in log variance) were classified by the SVM ($C=1$). They were able to achieve a mean classification accuracy of 72% (with $N=2$). We adapted their analysis by classifying $N \in \{2, 4, 6, 8, 10\}$ number of CSP components in the same temporal and frequency window.

**Fig. 7:**
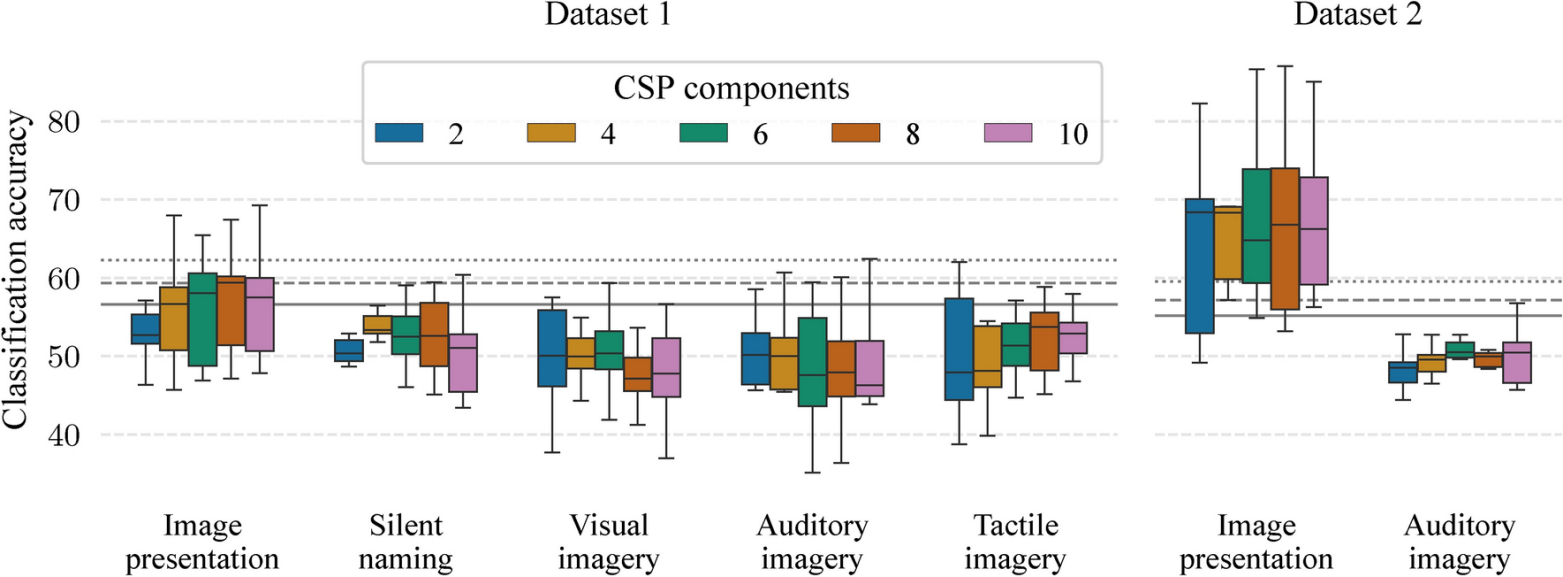
Mean classification accuracies from adapted analysis^22^ in which the SVM (CV) can utilize a certain number of CSP components. Horizontal lines represent significant borderlines for $p=0.05$ (solid), $p=0.01$ (dashed), and $p=0.001$ (dotted).

Figure 7 shows the results of this adapted analysis using the SVM with the C parameter selected by nested-cross-validation. Mean classification accuracies for the image presentation were significant only for 8 and 10 CSP components ($p < 0.05$) in Dataset 1. In Dataset 2, mean accuracies were between 63.7% ($p < 10^{-5}$)–67.5% ($p < 10^{-6}$). For all of the mental task periods, mean classification accuracies were not statistically significant in both datasets. The average number of participants with significant accuracies across all classifiers in the image presentation was between 2.9–6.1 across different number of CSP components in Dataset 1 and between 4.1–7.0 in Dataset 2. In the mental tasks, these average number were between 0.0–2.0, 0.0–2.3, 1.0–1.7, and 0.3–2.0 for the silent naming, visual imagery, auditory imagery, and tactile imagery task in Dataset 1, respectively, and 0.0–1.0 for the auditory imagery task in Dataset 2.

Overall, both analyses revealed the possibility of differentiating between the semantic categories only during the image presentation period.

## Discussion

Our results indicate a potential issue when semantic decoders are allowed to use neural activity recorded during the cue presentation period when moving toward semantic BCIs. We used state-of-the-art decoding analyses and further adapted analyses from two EEG-based semantic decoding studies to attempt to differentiate between the semantic categories of animals and tools during the image presentation and mental task periods.

Semantic decoding was possible during the image presentation period in all analyses. Mean classification accuracies were up to 69.9% when decoders could utilize information from all channels and all time points of the image presentation period. The sliding window approach revealed that the most informative period was between 54.7 and 429.7 ms after the image onset with the mean accuracy peak at the temporal window of 164.1–265.6 ms. Both of the two adapted analyses also allowed semantic decoding during the image presentation period with mean classification up to 71.3%.

However, *semantic decoding was not possible during any of the mental tasks across all participants in any of the analyses*. This is a more challenging problem for semantic BCIs, based on purely imaginary mental tasks without any external cue, than suggested by the current EEG-based semantic decoding studies. Furthermore, if the image presentation period had not been separated from the mental task period or if neural activity recorded during the image presentation period had been included with neural activity recorded during the mental task period, we could have claimed the possibility of semantic decoding with our presented analyses. A few participants had significant individual classification accuracies during some mental tasks and some classifiers, even though the semantic decoding was not possible during any of the mental tasks across all participants. In comparison, the majority of participants had significant individual accuracies during the image presentation period in all classifiers in both datasets.

The decoding performance differed between Datasets 1 and 2 when using the image presentation period. We believe there are several reasons for this. First, Dataset 2 had more repetitions per concept resulting in more trials per category to train the decoders (126 trials per category versus 90 trials in Dataset 1). Second, participants in Dataset 2 were members of our lab. Thus, there could be a difference in participants’ motivation and engagement between the two datasets. Nevertheless, even these changes did not improve decoding performance for any of the mental tasks.

Perceiving an external cue and performing a mental task elicit complex neural activity that comprises, but is not limited to, perceptual processes, semantic activation caused by perception, and semantic activation caused by concept-related mental tasks. Thus, decoders might exploit the perceptual processing-related neural activity during the image presentation period. However, we do not believe that the semantic decoding performance is entirely based on perceptual processing while perceiving the image. If this were the case, we would expect to see mainly channels with significant accuracies from the brain areas related to perceptual processing. This is visual processing in our case and thus we would expect to see primarily channels from occipital areas. However, the decoders were able to utilize channels from all areas, not limited to occipital areas. Additionally, channels with maximal accuracies were not from occipital areas. The combination of motor, parietal, and frontal areas was heavily utilized by our decoders. This is especially clear on Dataset 2 (see Figures 3 and 5).

In the silent naming tasks, Murphy et al.^22^ reported that a wide range of occipital, parietal, and frontal areas played a role in separating the semantic categories of mammals and tools in EEG. Soto et al.^38^ reported significant decoding of semantic categories in fMRI in the middle temporal gyrus, anterior and inferior temporal, inferior parietal lobe, and prefrontal regions. Nevertheless, prefrontal regions are typically believed to be involved in semantic control rather than in representing semantic knowledge^39–41^.

Our imagery mental tasks differ from the mental tasks examined in previous EEG-based semantic decoding^1^. Instead, our mental tasks should rather be compared with the results from mental imagery research^42–47^. A review by McNorgan^43^ found a general imagery network shared between different modalities. The review surveyed auditory, tactile, motor, gustatory, olfactory, and visual imagery in fMRI and PET studies. Shared activations between modalities (regardless of mental tasks) were found in bilateral dorsal parietal, left inferior frontal, and anterior insula regions. Modality-specific imagery for most modalities was also associated with activations in corresponding sensorimotor regions (but not necessarily in primary sensorimotor areas), primarily left-lateralized.

Our imagery mental tasks did not attempt to constrain the imagery strategies used by the participants in any way. Although our participants were directed to use a particular sensory modality (e.g., tactile modality), we did not restrict participants to use only this modality. We allowed them to use anything that came naturally to them. Many participants reported using visual imagery in other imagery tasks. The current evidence suggests that the early visual cortex is involved in visual imagery when the imagery task requires high-fidelity representations^43,48^.

Only a few semantic decoding studies attempted semantic decoding during a free recall paradigm with minimal cue support^29,49,50^. During free recall, participants are often instructed to recall semantically related items consecutively. This is the most similar paradigm to our experimental design. For instance, a fMRI study^49^ presented participants with semantic categories of animals and tools in four modalities: spoken words, written words, images, and natural sounds. Participants were asked to respond upon appearance to out-of-category exemplars. Additionally, the experiment ended with two free recall blocks in which participants were presented with a category name for 2 seconds and then instructed to recall all the entities from the cued category, seen during the experiment, in 40 seconds. A classifier was trained on the data from the actual stimulus presentation and then tested on the recall data. Mean classification accuracies across 14 participants were significant only when the classifier was trained on a combination of all modalities (67%) and partly when trained on a natural sounds modality (65%, $p=0.01$, but below the *p*-value threshold for statistical significance used by the study). In general, studies that examined neural activity during free recall showed the rise and fall of the category-specific neural activity with different effects in different neuroimaging modalities^29,50–55^.

No exploratory research can ever be completely comprehensive and we may have neglected to include some methods and approaches. We selected the current state-of-art analyses from prior EEG-based semantic decoding studies. It is important to note that the majority of our analyses considered primarily the temporal domain. This inherently assumes a time-locked neural activity to a certain degree. A high trial variability could explain the inability to achieve significant semantic decoding during the mental tasks. This would be especially the case for Dataset 1 in which participants were required to quickly switch between different mental tasks with only a 200 ms gap between the mental tasks. To avoid this issue, Dataset 2 used only one mental task with even longer periods for the image presentation and the mental task. However, this change in Dataset 2 did not improve the decoding performance. Our observations from other more complex analyses of these datasets^56^, which are not reported here, suggest the need for (non-phase-locked) time-variant approaches due to considerable variability in temporal and spectral locations of statistically significant classification accuracies across participants and mental tasks. Examples of these methods are, for instance, recurrent neural networks such as long short-term memory networks^57,58^. On the other hand, responses to stimuli often exhibit a relatively high signal-to-noise ratio (SNR) compared to responses to other cognitive events. For instance, event-related potentials (ERPs) typically have a higher SNR than event-related desynchronization/synchronization (ERD/S)^59^. Furthermore, the cue-related neural activity is time-locked to the cue presentation, allowing for more effective averaging over trials compared to non-stimuli-locked events, which makes decoding easier.

We used a successful semantic decoding approach from prior studies to attempt to differentiate between the two most used semantic categories of animals and tools. Nevertheless, this broad characterization of the semantic categories could pose greater challenges when the decoding problem becomes more intricate, that is when the cue presentation and mental task periods are separated. A prediction of individual concepts might lead to better results. This is typically achieved by training a model that can predict coordinates in some feature space that can be then transformed into individual concepts. The idea is that semantically similar concepts would be located closer to each other in that feature space. There are many different approaches to model feature spaces^1,60^. Additionally, this approach allows for generalization by zero-shot learning to predict items a model has not been trained on.

While we purposely avoided utilizing neural activity recorded during the cue presentation period for decoding purposes, experimental designs with separate blocks for only training semantic decoders could be used. In these experimental designs, neural activity evoked during the cue presentation period could be utilized to train semantic decoders, which might improve decoding performance during subsequent blocks when the cue period is no longer utilized. This approach would be based on the reinstatement of neural activity between encoding and retrieval from memory retrieval research^29,61–63^. Despite this, recent findings indicate that even separating the cue presentation before the visual imagery task can influence decoding performance^64,65^. Specifically, a study^64^ demonstrated that visual imagery tasks preceded by either a visual cue (an image of the object) or an auditory cue (a spoken name of the object) affect outcomes differently. The authors argue that the paradigm when visual cues preceded the visual imagery tasks captures short-term retention in visual working memory^66–68^ rather than spontaneous mental imagery, which is necessary for an actual BCI application. They showed that while decoding the four image categories was possible during the visual imagery task following the visual cue, decoding was not possible during the visual imagery task following the auditory cue. Our study differs in two main aspects: their visual imagery task followed the image presentation immediately, while ours included a gap; and our visual imagery tasks were unconstrained, allowing participants to visualize their own representation of the presented concept, whereas their tasks required participants to vividly recall the specific image. Overall, these differences underscore the current challenges in developing effective imagery-based tasks for BCIs.

## Methods

### Participants

#### Dataset 1

Twelve right-handed native English speakers were recruited from the student and staff population of the University of Essex (3 males and 9 females, age range 20–57, mean 32.75, standard deviation 11.55). The recruitment search was limited to native English speakers to avoid potential differences in neural representations of semantic concepts by individuals who speak different languages^69,70^. This restriction was done primarily for the silent naming task. All participants had normal or corrected-to-normal vision and gave written informed consent prior to the experiment. Participants received compensation of £16 for their time. The research protocol was approved by the Ethics Committee of the University of Essex on 25th October 2018. The experiment was performed in accordance with relevant guidelines and regulations. This experiment was also described in^71^.

#### Dataset 2

Seven participants were recruited from members of our lab (5 males and 2 females, age range 27–44, mean 34, standard deviation 5.98). All participants were right-handed and had normal or corrected-to-normal vision. None of the participants were native English speakers. This constraint was not required for this dataset because this experiment did not contain the silent naming task. All participants gave written informed consent before the experiment. The research protocol was approved by the Ethics Committee of the University of Essex on 20th February 2023 (ETH2223-0805). The experiment was performed in accordance with relevant guidelines and regulations.

### Tasks

Participants were instructed to perform a set of four individual mental tasks (silent naming, visual, auditory, and tactile imagery) after they were presented with images from two semantic categories of animals and tools. The order of mental tasks was randomized across blocks.

In the silent naming task, participants were instructed to name the presented object in their minds (in English). In the visual imagery task, participants were instructed to visualize the presented object, but using their representation of the concept instead of the particular image they had seen. In the auditory imagery task, they were instructed to imagine sounds made by the presented object when they were interacting with it. For instance, the sounds made by an animal (such as the mewing of a cat) or the sounds produced when using a tool (such as the banging of a hammer). Lastly, in the tactile imagery task, participants were instructed to imagine the feeling of touching the presented object. For instance, when petting an animal or touching different parts of a tool. In addition to the above-mentioned descriptions and examples of the mental tasks, participants were instructed to use the imagery strategy that came most naturally to them. For all imagery tasks, they were instructed to be engaged for the whole mental task duration (3 seconds).

#### Dataset 2 differences

Only the auditory imagery task was used (for 5 seconds).

### Stimuli

Images of 18 animals and 18 tools were sourced from the Internet. We selected the pair of semantic categories and the visual modality (images) to cue participants because they were the most used in the semantic decoding studies^1^. Images were converted to gray-scale, cropped, resized to $400 \times 400$ pixels, and contrast stretched. The object was presented on a white background. The selected concepts are listed below.

Animals: bear, cat, cow, crab, crow, dog, donkey, duck, elephant, frog, lion, monkey, owl, pig, rooster, sheep, snake, and tiger.

Tools: axe, bottle-opener, broom, chain saw, computer keyboard, computer mouse, corkscrew, hammer, hand saw, hoover, kettle, knife, microwave, pen, phone, scissors, shovel, and toothbrush.

### Experimental design

Figure 1 shows an illustration of a concept trial. Each concept trial started with a black fixation cross on a white background for 1–2 seconds (uniformly distributed). The image of a concept was then presented for 0.6 seconds. A mask (the image of a checkerboard) followed for 0.6 seconds to reduce visual persistence and thus to get rid of potential effects of perceptual processing related neural activity from the image presentation after this mask presentation^72^. A blank white screen was shown for 0.5 seconds before a sequence of all four mental tasks. Each mental task lasted for 3 seconds and was separated from the following one by a blank white screen for 0.2 seconds. The type of the mental task was indicated by text presented on the screen for the whole mental task duration: “Silently name”, “Visualize”, “Listen”, or “Feel”. After the last task, a short break for 2 seconds was given to participants in which a blank screen was presented, which changed color over time from white to black and back. In total, the presentation of one concept took 17.3–18.3 seconds.

Each concept was presented five times, that is for a total of 90 trials per category (18 concepts, 5 repetitions each). The experiment contained 15 blocks with 12 concepts per block (207.6–219.6 seconds). Each block was followed by a break of at least 30 seconds. There was one longer break of at least 3 minutes in the middle of the experiment. Additionally, the experiment started with two short blocks (86.5–91.5 seconds, a random subset of 5 concepts, each repeated two times) for familiarization with the experiment, that are excluded from the analysis.

The order of concepts and mental tasks was pseudo-randomized. No concept was repeated twice in succession. All mental tasks in one block had the same order. Different blocks had different orders of mental tasks while no order in a given block was repeated in the following block.

#### Dataset 2 differences

The experiment used to record Dataset 2 was a simplified variant of the experiment used to record Experiment 1 and used only the auditory imagery task (see Fig. 1). The cue presentation period of the concept image was extended to 1 second, the duration of the auditory imagery task was extended to 5 seconds, and the blank screen period before the mental task was changed to 0.5–1 second (uniformly distributed). In total, the presentation of one concept took 10.1–11.6 seconds. Each concept was presented seven times and, thus, the total number of trials per category was increased to 126 (18 concepts, 7 repetitions each). The experiment contained 14 blocks with 18 concepts per block (181.8–208.8 seconds). The breaks between blocks were the same as in the first experiment. The order of concepts was pseudo-randomized as in the first experiment, so as not to repeat any concept twice in succession, with an additional constraint that the semantic categories are balanced in each block.

### Data acquisition

EEG data were acquired with a BioSemi ActiveTwo system with 64 electrodes positioned according to the international 10–20 system, plus one electrode on each earlobe as references, with a sampling rate of 2048 Hz.

### EEG data preprocessing

The EEG signals, from both datasets, were referenced to the mean of electrodes placed on the left and right earlobe. Channels with bad signal quality were manually identified and removed. The EEG data were high-pass filtered to remove slow drift artifacts by an IIR 4th-order Butterworth filter with a 1 Hz cutoff (forward-backward filtering, sosfiltfilt method from SciPy^73^). Epochs representing concept trials (a presentation of a concept image with a sequence of all mental tasks) were extracted from the last second of the fixation cross, before the image presentation, until the end of the last task (14.3 seconds after the image onset in Dataset 1 and 7.1 seconds in Dataset 2). We inspected the concept trials for artifacts in any channel and marked bad concept trials that were then excluded from further analysis. Participant 8 in Dataset 1 was excluded from further analysis due to overall bad signal quality and artifacts in most concept trials.

Artifacts from eye blinks were suppressed by independent component analysis (ICA)^74,75^. Spatial ICA (FastICA from SciPy^73^ with default settings except with a maximum number of iterations set to $10^4$ and with a number of components corresponding to a number of valid channels) was fitted on the valid concept trials for each participant. One component from the ICA that represented eye blinks was removed from the high-pass filtered data.

### Analysis in the temporal domain

The analysis in the temporal domain was conducted in the two most widely used frequency bands (see Table 1): 1–30 and 4–30 Hz. In both cases, the preprocessed EEG data were first high-pass filtered by an IIR 4th-order Butterworth filter (1 or 4 Hz cutoff, forward-backward filtering, sosfiltfilt method) and then convolved with a FIR low-pass filter (designed by the firwin method from SciPy^73^, 0.661 s length, 30 Hz cutoff, 5 Hz bandwidth)^76^. Lastly, the filtered data were downsampled to 128 Hz and restructured into epochs of interest (excluding data from the excluded concept trials).

Two approaches for the classification were investigated: a sliding window approach and the classification of the whole period of interest. The sliding window approach was used to investigate the temporal evolution of semantic decoding. A temporal window of 109.375 ms was used (14 time points at a sampling frequency of 128 Hz) with a step of half the window size. A classifier was separately trained in each position of the temporal window by using only time points from that window. In the classification of the whole period, a classifier could use information from all time points in the period of interest.

In both approaches, data in each temporal window were normalized (z-scored) for each channel separately (based on training folds during cross-validation) and a classifier was trained in a stratified 15-fold cross-validation. We used these analyses on each channel separately and on all channels.

We used the following classifiers (all from scikit-learn^77^ with default settings unless specified): the SVM with the radial basis function kernel and $C=1$, LR with L1 or L2 norm (with a maximum number of iterations set to $10^5$), LDA, and the RF classifier. Additionally, we used the SVM with a value for the *C* parameter chosen by a nested cross-validation, the LR with L1 norm with a regularization strength parameter value selected by a nested cross-validation, and the RF with a nested cross-validation search for a number of trees in the forest from {10, 100, 200, 500} and a maximum depth of the tree from {5, 10} or unlimited. In all instances, a stratified 10-fold inner cross-validation was used.

Decoding performance was measured by classification accuracy. Statistical significance for the mean classification accuracy across participants was calculated using a bootstrapping simulation (based on $10^6$ simulations using a classifier that randomly chooses a class). Significance borderlines were then 56.6% for $p = 0.05$, 59.33% for $p = 0.01$, and 62.25% for $p = 0.001$ in Dataset 1 and 55.16%, 57.14%, and 59.52% respectively in Dataset 2.

### A comparison to state-of-the-art

We adapted the analysis from a study by Simanova et al.^21^ in the following pipeline. The preprocessed EEG data were filtered in the frequency band of 1–30 Hz and the first 600 ms were extracted from periods of interest. Each channel was separately normalized (z-scored). A classifier used all channels for the classification. We tested all classifiers as in the previous section. Note that the LR with L1 norm is similar to the approach reported in^21^ (but without explicitly using a Bayesian approach). Because our image presentation in Dataset 1 was only 600 ms, in comparison with their 700 ms, we used a temporal window of 600 ms for all classified periods.

We adapted the analysis from a study by Murphy et al.^22^ in the following pipeline. The preprocessed EEG data were filtered in the frequency band of 4.1–18.3 Hz and epochs were extracted at the temporal window of 95–360 ms. We performed dimensionality reduction by principal component analysis (PCA) before the CSP step to avoid the issue of decreased dimensionality of EEG signals after ICA for eye blinks suppression during the preprocessing phase^78^. The number of dimensions was decreased by one because one IC was removed. Then data were spatially filtered by CSP and a subset of *N* CSP components were extracted. The first half of *N* selected CSP components had the largest eigenvalues and the second half had the smallest eigenvalues. The log variance of the selected CSP components was classified by all considered classifiers. We tested $N \in \{2, 4, 6, 8, 10\}$.

## Data Availability

The data that support the findings of this study are available on request from the corresponding authors [M.R. and I.D.]. The data will be publicly available after the end of the ongoing project.

## Code availability

The code that supports the findings of this study is available at https://github.com/milan-rybar/semantic-neural-decoding-cue.
